# Paternity in wild ring‐tailed lemurs (*Lemur catta*): Implications for male mating strategies

**DOI:** 10.1002/ajp.22584

**Published:** 2016-07-08

**Authors:** Joyce A. Parga, Michelle L. Sauther, Frank P. Cuozzo, Ibrahim Antho Youssouf Jacky, Richard R. Lawler, Robert W. Sussman, Lisa Gould, Jennifer Pastorini

**Affiliations:** ^1^ Department of AnthropologyCalifornia State University‐Los AngelesLos AngelesCalifornia; ^2^ Department of AnthropologyUniversity of Colorado at BoulderBoulderColorado; ^3^ Department of AnthropologyUniversity of North DakotaGrand ForksNorth Dakota; ^4^ Département des Sciences BiologiquesUniversité de ToliaraToliaraMadagascar; ^5^ Department of Sociology and AnthropologyJames Madison UniversityHarrisonburgVirginia; ^6^ Department of AnthropologyWashington UniversitySt. LouisMissouri; ^7^ Department of AnthropologyUniversity of VictoriaVictoriaBritish ColumbiaCanada; ^8^ Anthropologisches InstitutUniversität ZürichZürichSwitzerland; ^9^ Centre for Conservation and ResearchRajagiriyaSri Lanka

**Keywords:** Bezà Mahafaly, extra‐group mating, Madagascar, reproductive skew, sexual selection

## Abstract

In group‐living species with male dominance hierarchies where receptive periods of females do not overlap, high male reproductive skew would be predicted. However, the existence of female multiple mating and alternative male mating strategies can call into question single‐male monopolization of paternity in groups. Ring‐tailed lemurs (*Lemur catta*) are seasonally breeding primates that live in multi‐male, multi‐female groups. Although established groups show male dominance hierarchies, male dominance relationships can break down during mating periods. In addition, females are the dominant sex and mate with multiple males during estrus, including group residents, and extra‐group males—posing the question of whether there is high or low male paternity skew in groups. In this study, we analyzed paternity in a population of wild *L. catta* from the Bezà Mahafaly Special Reserve in southwestern Madagascar. Paternity was determined with 80–95% confidence for 39 offspring born to nine different groups. We calculated male reproductive skew indices for six groups, and our results showed a range of values corresponding to both high and low reproductive skew. Between 21% and 33% of offspring (3 of 14 or three of nine, counting paternity assignments at the 80% or 95% confidence levels, respectively) were sired by extra‐troop males. Males siring offspring within the same group during the same year appear to be unrelated. Our study provides evidence of varying male reproductive skew in different *L. catta* groups. A single male may monopolize paternity across one or more years, while in other groups, >1 male can sire offspring within the same group, even within a single year. Extra‐group mating is a viable strategy that can result in extra‐group paternity for *L. catta* males.

## 1 INTRODUCTION

Reproductive skew refers to the distribution of reproductive success among same‐sex individuals in a population (Clutton‐Brock, 1998). Among males, several factors can determine the extent of reproductive skew. For example, high male reproductive skew is expected where female fertile periods do not overlap and where stable male dominance hierarchies determine mating priority (Altmann, 1962). Conversely, male reproductive skew is expected to be lower where there are more male competitors (Kutsukake & Nunn, 2006), greater numbers of females (Kappeler & Port, 2008), or where overlap in female receptive periods disrupts the ability of single males to monopolize mating opportunities (Ostner, Nunn, & Schulke, 2008).

Apart from its interesting behavioral causes and consequences, reproductive skew is important to document for its influence on microevolutionary dynamics. When reproduction is concentrated to a limited number of males within a population (assuming an equal sex ratio), skewed reproduction ultimately lowers the variance effective size of a population (NeV), thereby increasing the effects of genetic drift; this is particularly relevant to protected and threatened populations. Further, reproductive skew has the potential to unite offspring cohorts at the half‐sib level (since infants all share the same father), thereby promoting the opportunity for kin selection to act (Altmann, 1979; Widdig, 2013). Finally, reproductive skew influences the opportunity for sexual selection, and thereby the evolution of sexually dimorphic traits (Shuster & Wade, 2003).

Among primates with multiple male residents per group, paternity can be highly skewed, with one or just a few males siring group offspring (e.g., capuchin monkeys, *Cebus capucinus*: [Jack & Fedigan, 2006; Muniz et al., 2010]; red‐fronted lemurs, *Eulemur rufifrons*: [Kappeler & Port, 2008; Wimmer & Kappeler, 2002]). Conversely, paternity may show relatively low skew, with reproductive success being distributed among several males (e.g., *Macaca assamensis*: [Sukmak, Wajjwalku, Ostner, & Schülke, 2014]) who may or may not be related. Related males siring infants in the same group would have effects similar to high paternity skew: increased relatedness of offspring cohorts that would favor kin selection (Altmann, 1979; Widdig, 2013). Sires can also come from within the group (residents) or outside of the group (extra‐group males).

Occasionally, extra‐group male parentage can be considerable, such as in Verreaux's sifaka, *Propithecus verreauxi*, where non‐residents sire between 17% and 65% of group offspring each year (Lawler, 2007; Lawler, Richard, & Riley, 2003). Similarly, in one population of langurs (*Semnopithecus entellus*), nonresident males sired 21% of the infants in multi‐male groups (Launhardt, Borries, Hardt, Epplen, & Winkler, 2001). Rhesus macaques, *Macaca mulatta*, on Cayo Santiago show extra‐group paternity rates as high as >59% in some years (Georgiev et al., 2016). Rates of extra‐group paternity are expected to be highest where resident males have greater difficulty monopolizing copulations with females (e.g., groups with a female‐biased sex ratio: [Lawler et al., 2003]).

In this study, we investigated male reproductive skew, extra‐group male paternity, and evaluated the relatedness of sires in the ring‐tailed lemur, *Lemur catta*, a seasonally breeding primate endemic to Madagascar that lives in multi‐male, multi‐female groups and is female dominant (Jolly, 1966; Kappeler, 1990; Pereira, Kaufman, Kappeler, & Overdorff, 1990; Sauther, Sussman, & Gould, 1999). This primate species is of unique interest because it is characterized by extremes in reproductive traits (i.e., asynchronous estrus that lasts for <1 day [Pereira, 1991; Sauther, 1991]) that would seem to favor male monopolization potential, but other aspects of this species’ behavior might be expected to promote low paternity skew. For example, established groups usually have a single high‐ranking male who is dominant over other group males (Sauther, 1991; Sauther & Sussman, 1993) and this alpha male tends to mate first and mate guard for longer periods than other males (but see Gould, 1994; Parga, 2003; Sauther, 1991). As such, paternity skew could be high, especially if a first‐mate fertilization advantage operates in this species (Pereira & Weiss, 1991). However, male dominance relationships can be highly unstable during mating periods (Gould, 1994, 1997; Gould & Ziegler, 2007; Jolly, 1966; Koyama, 1988; Parga, 2009), and females (due to their social dominance) exercise a high degree of mate choice (Gould, 1994; Koyama, 1988; Pereira & Weiss, 1991; Sauther, 1991; Taylor, 1986; Taylor & Sussman, 1985), limiting the monopolization ability of high‐ranking males. Consequently, females mate with multiple males from within and outside of the group (Gould, 1994; Koyama, 1988; Sauther, 1991; Sussman, 1992), which should be expected to decrease reproductive skew. Clearly, the level of male reproductive skew or extent of extra‐group paternity is impossible to predict solely with behavioral measures, necessitating genetic analysis to address these issues.

## 2 METHODS

### 2.1 Study site and sample collection

Across seven capture years between 1987 and 2006, we collected 243 biological samples (blood or hair) from safely captured, sedated *L. catta* belonging to groups in and around the gallery forest portion of the Bezà Mahafaly Special Reserve (BMSR), Madagascar. Beginning in 2003, all groups in and surrounding Parcel 1 of BMSR were sampled. The lemurs were studied as part of long‐term research on *L. catta* ecology, health, behavioral endocrinology, and demography (Gould, Ziegler, & Wittwer, 2005; Gould & Ziegler, 2007; Sauther et al., 2006; Sussman & Ratsirarson, 2006). These samples derived from 14 distinct *L. catta* groups, each with 3–8 males and 2–9 females. Beginning in 2003, yearly records were kept of membership within reserve groups. Records of group membership in BMSR prior to this date were made opportunistically. For further details on methodology, including capture protocol, refer to Sauther et al. (2006) and Parga, Sauther, Cuozzo, Youssouf Jacky, and Lawler (2012). Since 2003, all animal handling was conducted with Institutional Animal Care and Use Committee (IACUC) approval from the University of Colorado and/or the University of North Dakota. This research also adhered to the American Society of Primatologists (ASP) Principles for the Ethical Treatment of Non‐Human Primates, and conformed to the legal requirements of the government of Madagascar.

### 2.2 Genetic analyses

The protocol for DNA extraction and amplification has been previously described (Parga et al., 2012, 2015; Pastorini, Fernando, Forstner, & Melnick, 2005, 2015). The following microsatellites were used: Lc5, Lc6, Lc7, Lc8, Lc9, Lc10 (Pastorini et al., 2005), 69HDZ267, 69HDZ299 (Zaonarivelo et al., 2007), Efr09 (Jekielek & Strobeck, 1999), Efr02 (Wimmer, 2000), L‐2 (Merenlender, 1993), Em7 (Pastorini, Fernando, Melnick, & Forstner, 2004), Em12 (Parga et al., 2015), and Pv1 (Lawler, Richard, & Riley, 2001). MICRO‐CHECKER, version 2.2.3 (van Oosterhout, Hutchinson, Wills, & Shipley, 2004) was used to evaluate the data for null alleles and scoring errors. One microsatellite (Lc9) showed evidence of scoring errors and null alleles, so was discarded. Approximately, 55% of samples were re‐genotyped from separate extractions to verify allelic data, as in Parga et al. (2012).

CERVUS (Kalinowski, Taper, & Marshall, 2007; Marshall, Slate, Kruuk, & Pemberton, 1998) was used to determine maternity and paternity via maximum likelihood methods for individuals born into the study groups who could be identified as natal offspring based on age. Fifty‐eight animals from 11 groups met the criterion of being natal offspring at the time of capture. Natal offspring were those that, at the time of capture, were either infants (1 yr), subadults (2 yrs), or young adults (3 yrs). All sampled adult males in the population (*N* = 141) were included as potential sires. Simulation parameters in CERVUS included 10,000 cycles, 72% of loci typed, and 188 candidate fathers (we assumed that the 141 males we sampled only represented 75% of possible sires). The “proportion of loci mistyped” was kept at the default value of 0.01. For every infant born, LOD (natural logarithm of the likelihood‐odds ratio) scores were calculated for each possible sire, indicating the likelihood that each male sired the offspring in question. The male with the highest LOD score was identified as the sire, excluding males who were too young at the time of infant conception to have sired offspring. Sire‐offspring pairs had to share at least one allele at each locus and were allowed no mismatching loci. Following standard convention, parentage assignments were made at two confidence levels: 80% and 95% (Marshall et al., 1998). Extra‐group paternity was determined when a male was identified as a sire but did not belong to the infant's social group at the time the infant was conceived, or by excluding all males residing in the group at the time of the infant's conception due to allelic mismatches at one or more loci.

As with high paternity skew, instances in which male kin sire same‐group infants can increase offspring relatedness (Altmann, 1979). We therefore used ML‐Relate (Kalinowski, Wagner, & Taper, 2006) to generate maximum likelihood estimates of pairwise relatedness (*r*) for males siring offspring within the same group in the same year. This program provides 95% confidence sets for relationships between pairs of individuals. For each set of male sires of same‐year and same‐group offspring, the most likely putative relationship between the two males (unrelated) was tested against the second most likely alternative (half‐sibling) using likelihood ratio tests, with a 0.05 significance level.

Methods to estimate pairwise relatedness and determine kin relationships from molecular data alone are not always accurate (Csillery et al., 2006; Van Horn, Altmann, & Alberts, 2008). Therefore, to test the predictive accuracy of ML‐Relate for our population, using CERVUS paternity and maternity assignments reaching 95% confidence, we created known sets of dyads in four different relatedness categories: parent‐offspring (*N* = 5), full‐siblings (*N* = 3), half‐siblings (*N* = 5), and unrelated individuals (*N* = 5). ML‐Relate identified the correct kin relationship (via a significant *P*‐value; data not shown) in four of five cases for parent‐offspring pairs (*r* = 0.5–0.67; mean *r* = 0.54), one of three cases for full‐siblings (*r* = 0.39–0.52; mean *r* = 0.48), two of five cases for half‐siblings (*r* = 0.27–0.57; mean *r* = 0.35), and one of five cases for unrelated individuals (*r* = 0.0–0.37; mean *r* = 0.13). Though ML‐Relate did not consistently perform well at hypothesis‐testing to determine proper kin relationships in our cohort of dyads who were known to be related, the *r* values generated by ML‐Relate were either at or above what would be expected for each relationship category (0.5 for parent‐offspring and full sib pairs, 0.25 for half‐sibs). Accordingly, whereas higher relatedness values (*r* ≥ 0.2) generated by ML‐Relate in this dataset cannot be consistently used to identify the proper kin category among relatives, low values of relatedness (*r* < 0.1) can be trusted in this dataset to identify unrelated individuals, as only dyads in our constructed cohort known to be unrelated show such low *r* values.

### 2.3 Determining reproductive skew

The software Skew Calculator 2013 (https://www.eeb.ucla.edu/Faculty/Nonacs/PI.html) was used to calculate Nonacs’ *B*, a binomial skew index (Nonacs, 2000, 2003) and to test whether the observed male reproductive skew per group was significantly different from a random distribution. The *B* index was calculated for groups producing more than one infant for which a sire could be identified at the 80% or 95% confidence level (*N* = 6) across the period spanning 2000–2005, which were the years across which the most complete paternity data for the greatest number of study groups was available. Groups for which such data were lacking (*N* = 7) were not used to calculate reproductive skew. This 6 year period was deemed appropriate for the calculation of the skew index because it falls within the time frame of some alpha male tenure durations at this location (e.g., in BMSR, three males were documented as having alpha tenures that lasted for 6 years [Sauther et al., 1999]). *B* index values closer to one indicate high reproductive skew, values closer to zero indicate low skew, and negative values indicate a more even distribution of paternity than would be expected by chance. *B* index values were calculated with 95% confidence intervals.

When calculating the *B* index, the Skew Calculator software is capable of incorporating data on male tenure for sires and non‐sires; therefore, we included data on the duration of male membership in each group (number of years each male was a group member) where such data were available, which was from 2003 to 2005 for most groups, with the exception of Green group, for which data on male group membership were available even earlier, beginning in 2001. Though male tenure data were not consistently available across all 6 years of the period of infant production we considered (2000–2005), we deemed it more accurate to include all available data on male tenure rather than exclude it, especially when such data were available for groups for at least half (or more, the case of Green group) of the period under consideration.

Nonparametric statistics were run in Statistica 12 (Stat Soft Inc., Tulsa, OK, 2013). In particular, Spearman rank correlation tests were used to determine whether aspects of group composition across the multi‐year period of analysis (the total number of group males, the average number of group males per year, the average number of group females per year) showed a significant relationship with the amount of paternity skew, as measured by the *B* index. Mann–Whitney *U* tests were used to determine if there was a significant difference in the number of males, number of females, or sex ratio between groups with and without extra‐group paternity. All tests were two‐tailed, with alpha set at 0.05.

## 3 RESULTS

**Figure 1:**
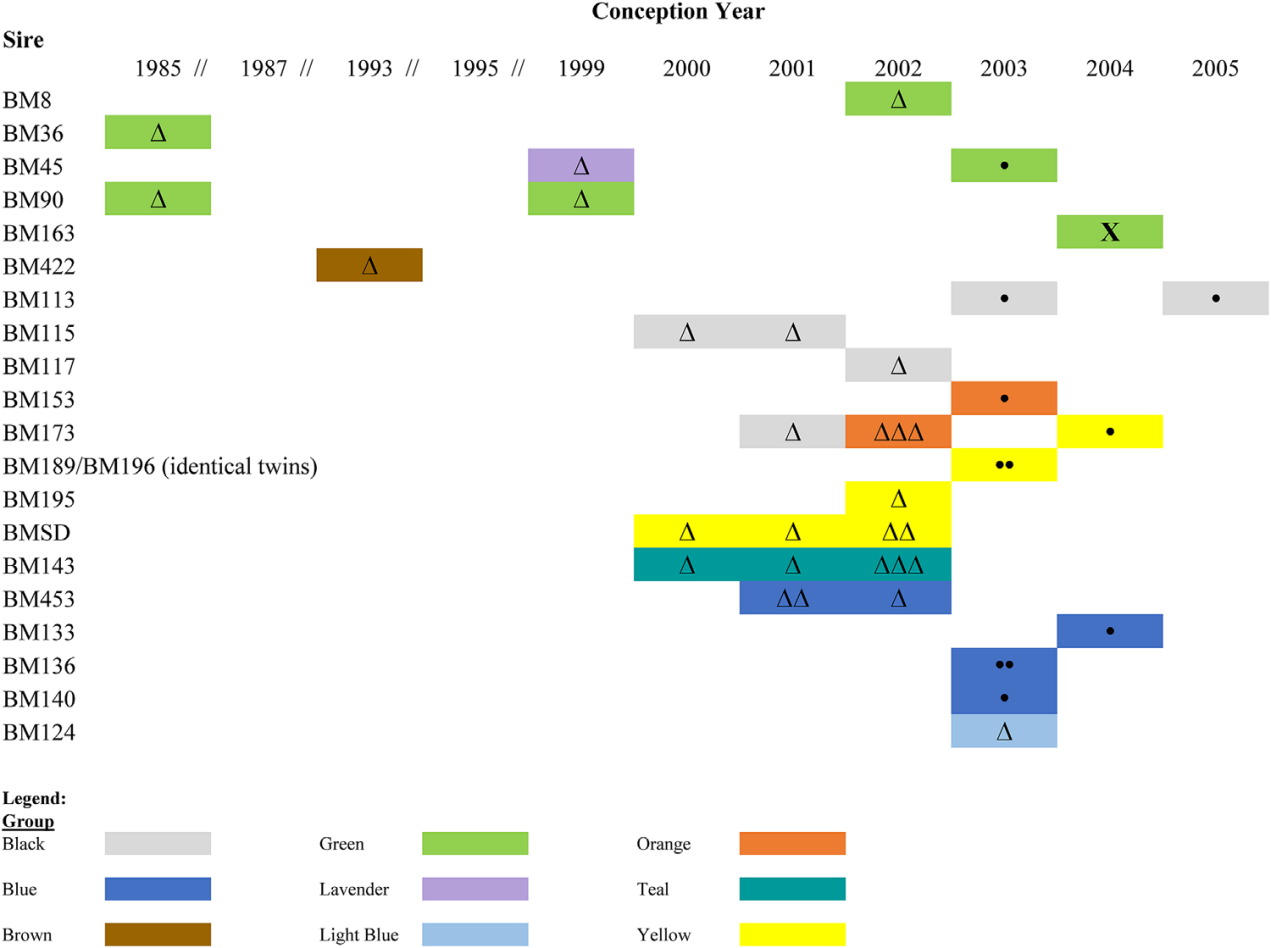
All paternity assignments made at the 80% and 95% confidence levels, indicating the number of offspring produced by each male. Each infant's group of birth is shown, indicating whether the sire was a resident (circles), whether male group membership was unknown at the time of conception (triangles), or whether the birth was a case of extra‐group paternity (X). Cases of extra‐group paternity where a sire could not be identified with at least 80% confidence, but where all residents were excluded as sires due to one or more allelic mismatches (*N* = 2), are not shown

Average heterozygosity and allele number were high for the microsatellite loci used in paternity analyses (Table 1). These loci had a combined exclusionary power of 0.9996 when assigning the first parent (both parents being unknown), and 0.9999 when assigning the second parent. Out of 114 males in the population, 21 males were identified as sires (including a pair of identical male twins who were genetically indiscernible) at the 80% confidence level. If strictly counting only assignments at the 95% confidence level, 17 males were identified as sires (including the identical male twins). Although sires produced between 1 and 3 infants per year, each sire only produced offspring within a single group per year (Figure 1).

**Table 1:** Heterozygosity and allele number for loci used in paternity analysis

Locus	*k*	H_O_	H_E_
Lc5	9	0.750	0.778
Lc6	8	0.750	0.734
Lc7	10	0.900	0.838
Lc8	7	0.733	0.757
Lc10	10	0.807	0.794
69HDZ267	10	0.800	0.816
69HDZ299	7	0.700	0.795
Efr02	10	0.741	0.758
Efr09	12	0.800	0.740
L‐2	12	0.850	0.825
Em7	5	0.588	0.621
Em12	17	0.850	0.864
Pv1	13	0.841	0.869
Average	10	0.778	0.784

*k*, number of alleles; H_O_, observed heterozygosity; H_E_, Nei's (1978) unbiased estimate of expected heterozygosity.

### 3.1 Paternity assignments and Nonacs’ *B* index

Male parentage could be assigned at the 80% or 95% significance level for 39 of the 58 offspring who were identified as natal at the time of capture. The 39 offspring came from nine different groups (Figure 1). Of the 19 infants for whom a sire could not be identified with ≥80% confidence, it appears that the sire may not have been sampled for nine of the infants, and for the remaining 10 infants, a sire was identified but the paternity assignment did not reach statistical significance. For all offspring for whom a sire could be identified at either the 80% or 95% significance level, the paternity assignment (which was based on the male with the highest LOD score) agreed with mother‐offspring‐father trio genotypes.

When testing for paternity skew, statistical significance was reached by three of the six groups for which Nonacs’ *B* index values were calculated (Table 2). Among these groups, one showed high skew (Teal, *B* = 0.734, 95% CI: [0.227–0.734], *P* < 0.0001), one group showed moderate skew (Orange, *B* = 0.378, CI: [0.046–0.794], *P* = 0.001), and a third showed low skew (Yellow, *B* = 0.158, CI: [−0.091–0.524], *P* = 0.049), although it should be noted that the confidence intervals for each show much overlap. In the group with the highest skew (Teal, Table 2), a single male was identified as the sire of five infants, siring three of those infants in a single year (Figure 1). In all other groups for which skew was calculated, ≥2 males sired group offspring, sometimes in the same year, sometimes in subsequent years (Figure 1). In the case of Yellow group, because the 95% confidence interval encompasses 0, the pattern of paternity observed was not significantly different from a random distribution of paternity among group males.

**Table 2:** Nonacs’ *B* index values per group and group parameters

Group	Total # known males in group during period	Ave # of males per year	Ave # of females per year	# Sires	# Infants	*B*	CI	*P*
Black	11	5.3	4.7	4	6	−0.039	−0.137–0.168	0.798
Blue	8	5	8.3	4	7	0.071	−0.116–0.496	0.156
Green	13	5.2	5	3	3	0.072	−0.282–0.539	0.290
Orange	10	5.3	6.3	2	4	0.378	0.046–0.794	0.001
Teal	14	7.7	5.3	1	5	0.734	0.227–0.734	<0.0001
Yellow	5	3.7	5.7	4	8	0.158	−0.091–0.524	0.049

Although these *B* index values were based upon paternity data for only those infants whose sires could be identified at the 80% confidence level, it is doubtful—as least for groups in which the *B* index values were statistically significant (Table 2)—that the inclusion of the additional infants for whom sires could not be identified would radically change the *B* index values. We suggest this because in each group, all or the majority of infants born had sires identified at the ≥80% significance level (Black: 6/6 infants; Blue: 7/10 infants; Green: 6/10 infants; Orange: 4/6 infants; Teal: 5/6 infants; Yellow: 8/10 infants). In the case of Teal group, if a different male was the sire of the remaining infant (for whom no sire is currently known), the *B* index value would slightly decrease, but would still be high, as one known male sired at least five of the group's six total offspring. In the case of groups with lower skew indices, identifying a greater number of sires per group would only further lower the already moderate‐to‐low skew indices. In short, our data would still show a wide range of *B* index values in different groups within this population if we had been able to identify an even greater number of sires at the ≥80% confidence level for infants in the study.

The *B* index did not show a significant correlation with the total number of group males across the period of analysis (Spearman: *r*_S_ = 0.26, *N* = 6, *P* = 0.62), average number of males per group per year (*r*_S_ = 0.38, *N* = 6, *P* = 0.46) or average number of females per group per year (*r*_S_ = 0.26, *N* = 6, *P* = 0.62). Indeed, there was substantial overlap in each of these variables in groups having both high and low reproductive skew (Table 2).

### 3.2 Extra‐group male parentage

Individual males only sired offspring within a single group per year, and both resident males and extra‐group males sired offspring. Among cases in which paternity assignments were made at the 95% confidence level, in three of nine (33%) cases, sires either did not belong to the social group at the time of conception (*N* = 1) or the true sire was unknown but all resident males were excluded as sires owing to ≥1 allelic mismatches with the offspring in question (*N* = 2). Counting paternity assignments made at the less conservative 80% confidence level, extra‐group male paternity was 21% (3 of 14 infants), with the same case of a single extra‐group sire known to be a resident in another group, and two inferred cases of extra‐group parentage. Admittedly, male group membership was unknown at the time of conception for many infants, so the actual values of extra‐group paternity measured here may be underestimated.

To test whether the number of resident males per group, the number of resident females, or the group sex ratio had a significant effect on extra‐group male parentage, a series of Mann–Whitney *U* tests were conducted on groups in which male and female membership data were available at the time of infant conception (*N* = 6). Although few groups showed extra‐group paternity, we found much overlap in the composition of groups with and without extra‐group paternity (Table 3). Neither the number of males (*U* = 11, *N*_1_ = 3, *N*_2_ = 8, *P* = 0.92), the number of females (*U* = 8.0, *N*_1_ = 2, *N*_2_ = 8, *P* = 1.0) nor the group sex ratio (*U* = 5.5, *N*_1_ = 2, *N*_2_ = 8, *P* = 0.53) showed a significant difference between groups with and without extra‐troop male paternity (Table 3).

**Table 3:** Comparison of groups with and without extra‐group paternity

Group	Conception year	# Males	# Females	Sex ratio	Extra‐group paternity?
Black	2003	3	5	0.6	No
Black	2005	8	4	2	No
Blue	2003	5	8	0.63	No
Blue	2004	5	9	0.56	No
Green	2003	7	6	1.17	No
Green	2004	5	6	0.83	Yes
Orange	2003	3	7	0.43	No
Orange	2004	6	6	1	Yes
Red	2002	4	Unknown	Unknown	Yes
Yellow	2003	5	5	1	No
Yellow	2004	4	6	0.67	No

### 3.3 Relatedness among sires

In three instances, two different males sired offspring in the same group during the same year (Figure 1, Table 4). In each instance, the sires appeared to be unrelated to one another, showing remarkably low estimates of relatedness (*r* = 0.0–0.04), although for only one pair did the test to evaluate the unrelated status of the males against the second most likely alternative (half‐siblings) reach statistical significance (Table 4). Nevertheless, all three estimates of relatedness between these “same group sires” were markedly lower than the relatedness values calculated for our cohort of male dyads known to be half‐siblings (*r* = 0.27–0.57; see Methods), which makes it unlikely that the “same group sires” shared any considerable degree of relatedness.

**Table 4:** Tests evaluating whether sires of same‐group offspring were significantly more likely to be unrelated than related at the half‐sibling level

Group	Conception year	Sires	*r*	*P*
Black	2001	BM 115 & BM 173	0.04	0.07
Blue	2003	BM 136 & BM 140	0.020	0.066
Yellow	2002	BM 195 & BM SD	0.0	0.009

*r*, estimated relatedness.

## 4 DISCUSSION

The *L. catta* groups in our population showed variability in male reproductive skew. One group showed considerable skew, with a single male siring >1 offspring in the same group in the same year as well as across multiple years. Another group showed more moderate skew (two sires of four offspring), while all other groups showed lower skew, with as many as four different males siring group offspring across a 6‐year period (sometimes with >1 sire in the same group in the same year). No consistent relationship was found between levels of paternity skew and group composition (average number of group males per year, average number of group females per year, or total number of males across the 6‐year period of analysis). Paternity skew is of interest because it is expected to favor kin selection via the creation of closely related cohorts of offspring; related males siring same‐group offspring would have a similar effect (Altmann, 1979). However, not only did most groups in our study show low skew, but different males who sired infants within the same group in the same year appeared to be unrelated.

When compared to other primates in which paternity skew has been measured using the *B* index, our *L. catta* groups showed a broad range and some of the highest skew values calculated among multi‐male primate groups. Multi‐male primate groups often show low or intermediate skew, with >1 male siring group offspring (e.g., northern muriqui, *Brachyteles hypoxanthus*, *B* = 0.012 [Strier, Chaves, Mendes, Fagundes, & Di Fiore, 2011]; rhesus macaques, *Macaca mulatta*, *B* = 0.0485–0.1068 [Dubuc, Muniz, Heistermann, Engelhardt, & Widdig, 2011; Georgiev et al., 2016; Widdig et al., 2004]; Assamese macaques, *M. assamensis*, *B* = 0.087: [Sukmak et al., 2014]). Half of our groups showed similarly low skew, with values approximating (or lower than) these published values. On the opposite end, our group with the highest skew—a single male siring offspring across a handful of years—exceeded published values of the *B* index for any primate to date. Even primates known to show high paternity skew (e.g., mountain gorillas (*Gorilla beringei*), *B* = 0.107–0.432 [Bradley et al., 2005]; white‐faced capuchins (*Cebus capucinus*), *B* = 0.083–0.473 [Muniz et al., 2010]) have *B* indices that are lower than that calculated for our group with the highest skew. However, we hasten to point out that this high skew is not the norm for our *L. catta* groups, and that the majority of our groups showed moderate to low paternity skew, comparable to those primate species in which reproduction is more equitably shared among males. Additionally, the confidence intervals for each group's skew index showed much overlap, likely because the *B* index is sensitive to sample size [Nonacs, 2000, 2003], and our sample size of sires and infants from which each of these skew values were calculated was small.

Although a review of the various reproductive skew models (Nonacs and Hager, 2011) is beyond the scope of this paper, between the two major sets of skew models—transactional, where dominants gain a benefit from the presence of subordinates and therefore “tolerate” their reproductive activity in the group (Keller and Reeve, 1994; Reeve, 2000) and “tug‐of‐war”/“limited control” (Clutton‐Brock, 1998; Johnstone, 2000; Reeve, Emlen, & Keller, 1998), where dominants are unable to keep subordinates from reproducing (e.g., mountain gorillas: Bradley et al. [2005])—*L. catta* appear to fall in the latter category. The aggressive competition that occurs among males during mating periods (Gould & Ziegler, 2007; Jolly, 1966; Koyama, 1988; Parga, 2006, 2009; Sauther, 1991) suggests that high‐ranking male *L. catta* are not conceding reproductive units to rivals (Reeve & Keller, 2001; Vehrencamp, 1983), but rather are unable to exclude other males from mating with females and siring group offspring. Indeed, in *L. catta* groups with established male dominance hierarchies, there is often a high‐ranking male who has first access to estrous females (but see Gould, 1994, 1996; Sauther, 1991), but this male is unable to monopolize mating with the estrous female (Koyama, 1988; Sauther, 1991). The tendency for *L. catta* females to mate multiply results in low male “mating skew” (Port & Kappeler, 2010) which in turn appears to translate into low paternity skew, the pattern evident for most groups in our study.

Extra‐group males in this study sired approximately 21–33% of offspring. This level of extra‐group paternity is comparable to that found in some other group‐living primates (i.e., langurs, *Semnopithecus entellus*: 21% [Launhardt et al., 2001], rhesus macaques, *Macaca mulatta*: 25–59% [Georgiev et al., 2016; Widdig et al., 2004], chimpanzees, *Pan troglodytes*: 0–10.5% [Boesch, Kohou, Nene, & Vigilant, 2006; Newton‐Fisher, Emery Thompson, Reynolds, Boesch, & Vigilant, 2010; Vigilant, Hofreiter, Siedel, & Boesch, 2001], Verreaux's sifaka, *Propithecus verreauxi*: 17–65% [but see Kappeler & Schaffler, 2008; Lawler, 2007; Lawler et al., 2003]). Thus, male visits to other groups during the breeding season (Gould, 1994; Sauther, 1991; Sussman, 1992) function as a viable mating strategy for *L. catta* males (Sauther & Sussman, 1993).

Extra‐group paternity is considered more likely to occur where group males experience difficulty monopolizing copulations with group females, such as where estrus periods overlap (Isvaran & Clutton‐Brock, 2007), or in groups having a female‐biased sex ratio (i.e., Verreaux's sifaka, *Propithecus verreauxi* [Lawler et al., 2003]). However, estrus synchrony is uncommon in *L. catta* (Pereira, 1991; Sauther, 1991), making this an insufficient explanation for extra‐group paternity in our population. Furthermore, neither the number of resident males per group, the number of females per group, nor group sex ratio was significantly different between groups with and without extra‐group paternity—though admittedly, our comparison is based upon very few instances (*N* = 3) of extra‐group paternity.

A contributing factor to extra‐group paternities is often female mate choice (Soltis, Thomsen, & Takenaka, 2001). Indeed, in species where extra‐group paternity is considerable (e.g., rhesus macaques, *Macaca mulatta*, with an average of 25% extra‐group paternity [Widdig et al., 2004] that can be as high as >59% in some years [Georgiev et al., 2016]), female choice has been implicated in the mating success of non‐resident males. Similarly, we suggest that our findings of extra‐group paternity and low skew within some groups is due to female mate choice for multiple males (Gould, 1994; Koyama, 1988; Parga, 2006; Pereira & Weiss, 1991; Sauther, 1991; Taylor, 1986) in this female dominant species (Jolly, 1966; Kappeler, 1990; Pereira et al., 1990; Sauther et al., 1999) coupled with resident males’ variable mate guarding activity (Parga, 2003, 2010; Sauther, 1991). Some *L. catta* males do not mate guard or mate guard for only minutes following ejaculation, whereas other males post‐copulatory guard for hours; none appears to mate guard throughout the female's entire estrus period (Parga, 2003, 2010; Sauther, 1991). The result is that females mate with both resident and extra‐group males (Gould, 1994; Koyama, 1988; Sauther, 1991; Sussman, 1992), with both types of males siring offspring (this study).

It is worth noting that very few of the males sampled in this study sired offspring (21 of the 141 males). Even if a sire had been identified for each infant for whom no sire was detected (or where paternity assignments did not reach statistical significance), the total number of sires (assuming a different male for every offspring), this would still only represent 28% (40/141) of males sampled. Hence, male reproductive skew *at the population level* may be considerable in *L. catta*. Admittedly, males who were not identified as sires may have sired offspring in the time period before or after this study, especially as males of this species frequently disperse between groups (which can include into and out of study areas) every few years (Koyama, Nakamichi, Ichino, & Takahata, 2002; Sussman, 1992). Furthermore, no male in this population sired offspring in >1 group per year, although some males sired >1 infant within a single group per year. Although we lack data on mating season dynamics, this pattern of paternity suggests that males may only be able to target a single group successfully during the mating season for reproductive activities—whether their own or another—but not both. This finding underscores the temporal limitations of male mating effort, and the difficulty for male primates of juggling competing activities while pursuing mating opportunities (Alberts, Altmann, & Wilson, 1996).

In conclusion, paternity skew in our *L. catta* population varied among groups, with some having high to moderate paternity skew, but most groups showing low paternity skew; non‐resident males also occasionally sired offspring. Still to be determined is the link between specific behavioral male mating strategies in this species and paternity success. Unfortunately, we did not consistently have male dominance rank data available for groups in the study, so whether high‐ranking males show greater reproductive success than other males is still an open question. Studies in non‐wild *L. catta* populations suggest that both dominant males and novel males (new immigrants) show paternity success (Pereira & Weiss, 1991; White et al., 2007), but more sampling is needed to determine whether such males have superior reproductive success in wild populations. Additionally, how mating dynamics differ in groups having high versus low paternity skew is currently unknown and is a topic for future research.
